# Combinatorial Control of mRNA Fates by RNA-Binding Proteins and Non-Coding RNAs

**DOI:** 10.3390/biom5042207

**Published:** 2015-09-24

**Authors:** Valentina Iadevaia, André P. Gerber

**Affiliations:** Department of Microbial & Cellular Sciences, Faculty of Health and Medical Sciences, University of Surrey, Guildford, Surrey GU2 7XH, UK; E-Mail: v.iadevaia@surrey.ac.uk

**Keywords:** gene expression, post-transcriptional control, RNA-binding protein, microRNA, ribonucleoprotein complex, combinatorial control

## Abstract

Post-transcriptional control of gene expression is mediated by RNA-binding proteins (RBPs) and small non-coding RNAs (e.g., microRNAs) that bind to distinct elements in their mRNA targets. Here, we review recent examples describing the synergistic and/or antagonistic effects mediated by RBPs and miRNAs to determine the localisation, stability and translation of mRNAs in mammalian cells. From these studies, it is becoming increasingly apparent that dynamic rearrangements of RNA-protein complexes could have profound implications in human cancer, in synaptic plasticity, and in cellular differentiation.

## 1. Introduction

The post-transcriptional control of gene expression is fundamental for proper cell homeostasis [1]. In particular, the fate of messenger RNAs (mRNAs) must be tightly regulated to prevent aberrant synthesis of proteins that could lead to anomalous development and eventual disease [1,2,3]. Regulation of mRNAs is thereby achieved by different RNA-binding proteins (RBPs) and non-coding RNAs, such as microRNAs (miRNAs), which interact with distinct elements in the mRNA, forming so-called ribonucleoprotein (RNP) complexes [4]. Different RNPs dynamically rearrange through the life cycle of an mRNA [4,5]. Upon synthesis of nascent transcripts by RNA polymerase in the nucleus, mRNA-precursors (pre-mRNAs) become immediately assembled with a host of proteins, which perform processing events such as capping, splicing, editing and polyadenylation [6]. Matured mRNAs are then exported to the cytoplasm by a variety of export factors and are localized to specific subcellular regions [7]. In the cytoplasm, mRNAs can be stored or captured by translation factors for assembly with ribosomes to serve as templates for protein synthesis. Thus, translation can be regulated by global means through translation initiation factors, while specific RBPs or miRNAs can modulate the control selected mRNAs [8]. Finally, transcripts are degraded through exonuclease-mediated degradation pathways and specific RBPs/miRNAs can direct specific degradation programs to subsets of mRNAs [4].

RBPs contain one or several characteristic RNA-binding motifs that specifically interact with RNA [5,9,10,11]. Some well-characterised RNA-binding domains refer to the RNA-recognition motif (RRM), the K-homology (KH) domain, the zinc finger motif (ZnF), the double stranded RNA-binding domain (dsRBD), and the Pumilio/FBF (PUF or Pum-HD) domain. Notably, RBPs often contain an array of the same or different RNA-binding motifs, which increases their specificity and affinity towards the RNA. Cytoplasmic RBPs preferentially interact with sequences or structural elements located in the 3'-untranslated region (3'-UTR) of mRNAs, allowing targeting of a subset of mRNAs to implement specific control. Importantly, the application of global analysis tools to comprehensively identify the mRNA targets for RBPs has revealed that RBPs preferentially bind to mRNAs coding for functionally related proteins—forming post-transcriptional operons or RNA regulons enabling coordinated control of mRNA expression [10,11,12,13].

mRNAs are also regulated via physical interactions with non-coding RNAs (ncRNA). Arguably, the best-characterised small ncRNAs (≤200 nts in length) are the microRNAs (miRNA), ~22-nucleotides (nts) long RNA molecules that negatively regulate gene expression in metazoans, with important roles in cancer development, progression and metastasis [14,15,16,17]. The production of miRNAs is complex and assisted by different RBPs [18]. Initially, miRNAs are transcribed as longer transcripts, so called primary miRNAs (pri-miRNA), which are then processed to precursor miRNAs (pre-miRNAs) involving the RNAse III protein Drosha. After export to the cytoplasm, pre-miRNAs are converted into mature miRNAs by Dicer (another RNAse III protein) and assemble with members of the Argonaute (Ago) protein family into the miRNA-induced silencing complex (miRISC). Of note, the maturation of particular miRNAs is further controlled by specific RBPs that bind to pri-miRNA or pre-miRNA sequences and thereby facilitate or block their maturation [18]. The miRISC complex then assembles with sequences located mostly in the 3'-UTRs of target mRNAs and induces changes in the subcellular localization, translation efficiency and stability [18]. The rules of miRNA-target recognition are still not fully understood yet. One important determinant relates to a requirement for perfect complementarity between the target site and 7–8 nucleotides at the 5'-end of the miRNA (region known as miRNA “seed”), while nucleotides further downstream (nucleotides 13–16) can also contribute to base pairing with the mRNA target [18,19]. In addition to miRNAs, an increasing number of long ncRNA (>200 nts) have also been recognized to regulate cytoplasmic translation events, such as the brain cytoplasmic (BC) family of lncRNAs, which regulate mRNA translation in neurons through interaction with translational initiation factors [20].

Bioinformatic searches have predicted more than one thousand RBPs (1542) [5], and almost double that number of miRNAs (2588) in the human genome [21]. Due to their vast numbers, it is not a great surprise that computational analysis predicted substantial combinatorial control of mRNA fates through simultaneous assembly of RBPs and miRNAs on particular mRNAs (e.g., [22]). Thus, looking at single proteins or the action of distinct miRNAs on mRNA fates alone could be misleading, as it does not consider the entire arrangement of *trans*-acting regulatory factors that affect specific mRNAs. Moreover, the differential expression of RPBs and miRNAs can lead to condition-specific assemblies restricted to particular cell-types or subcellular compartments. In this review, we discuss recent examples that support the notion of extensive combinatorial post-transcriptional control of mRNA stability and of translation through interactions with RBPs and/or miRNAs. We focus on recent mechanistic studies exemplifying antagonistic or synergistic arrangements of: (i) RBPs and miRNAs; (ii) RBPs and RBPs and (iii) miRNAs on cytoplasmic mRNAs and their impact in cell biology.

## 2. Cross-Talk between RBPs and miRNAs on Cytoplasmic mRNAs

An expanding number of reports demonstrate the interplay between miRNAs and RBPs on target 3'-UTRs under specific conditions. We highlight here some of the examples with direct implications in oncogenesis or cell differentiation, and the reader is referred to recent reviews highlighting additional studies [19,23,24,25]. Notably, most examples reported to date involve HuR, a member of the embryonic lethal abnormal vision (ELAV) family of proteins, which have wide-ranging roles in stabilizing mRNAs in the cytoplasm by binding to AU‑rich elements (ARE) preferentially located in the 3'-UTRs [26]. Whereas HuB, HuC, and HuD are neuronal or gonadal proteins, HuR is ubiquitously expressed and mediates cellular responses to different types of stress and coordinates inflammatory responses. HuR has been correlated with tumorigenesis in different cancer types, like breast, ovarian, colon, lung, and prostate cancer and in mesothelioma [27,28,29,30,31,32,33,34]. Moreover, HuR becomes predominantly cytoplasmic in certain tumour cells and correlates with decreases in patient survival rates [27].

**Figure 1:**
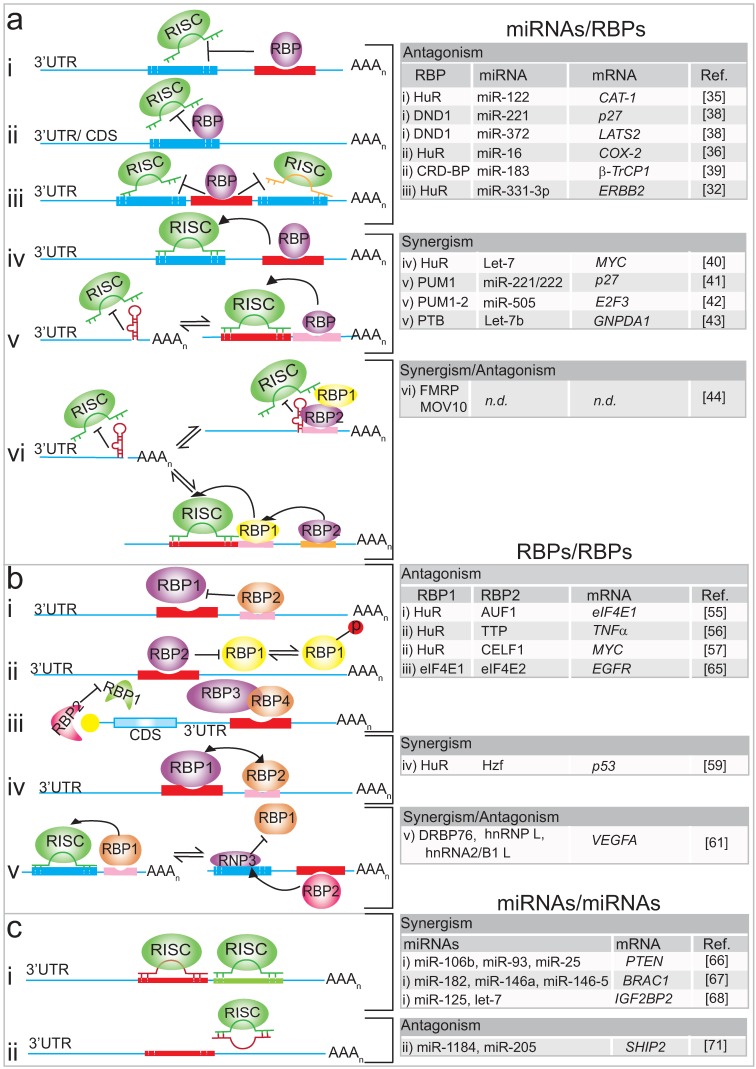
(**a**) Schematic of crosstalk between miRNAs and RBPs. Upper panel: The antagonistic interplay between RBPs with a mRNA target prevents miRNAs from binding to the transcript. RBP binding can occur next (i) to the miRISC binding site, in between (iii), or overlap in 3'-UTRs or coding sequences (CDS) (ii). Middle panel: Cooperation achieved by RBPs that promote/enable miRISC binding to mRNA targets (iv), thereby altering local RNA structures (v). Lower panel: Antagonistic and synergistic interplay between RBPs, where the miRISC activity on the same mRNA is determined by different combinations of RBPs; (**b**) Scheme of crosstalk between RBPs and RBPs. Upper panel: Competition of two RBPs for distinct (i) or the same (ii) RNA binding site. Translation initiation factors competing for common feature on the mRNA (cap) (iii). Middle panel: Cooperation between RBPs that promote mRNA export and translation (iv), Lower panel: Complex antagonistic and synergistic interplay between RBPs exemplified on VEGFA mRNA (v); (**c**) Scheme of crosstalk between miRNAs-miRNAs. Upper panel: Cooperation between different miRISC to enhance the inhibition of expression of the mRNA target (i). Lower panel: Competition between different miRISC to enhance the expression of the mRNA target (ii).

Several examples relate to the competition between an RBP and a miRNA for a particular binding site on an mRNA, resulting in antagonistic effects (Figure 1a (i–iii)). First described by Filipowicz and colleagues, HuR can relieve cationic amino acid transporter 1 (*CAT1*) mRNA from miR-122 mediated repression under stress conditions in human hepatoma carcinoma cells [35]. Upon amino-acid starvation, which triggers a cellular stress response, nuclear HuR is dephosphorylated and it translocates to the cytoplasm, where it binds to an ARE element in the 3'-UTR of *CAT-1* mRNA and thereby relieves the inhibition exerted by miR-122. HuR then stabilizes the *CAT-1* mRNA and enhances its translation by redirecting the mRNAs from processing (P) bodies (where mRNAs are degraded) to polysomes for protein synthesis (Figure 1a (i)) [35]. However, whether this is accompanied by the dissociation of miRNPs from the mRNA or just prevents miRNPs from acting as effectors in the repression remains to be established [35]. In colon cancer cells the expression of mRNAs coding for the pro-inflammatory enzyme cyclooxygenase-2 (COX-2) is controlled by direct competition between HuR and miR-16 for an overlapping binding site located in the 3'-UTR of the mRNA [36]. COX-2 is a key enzyme that converts arachidonic acid to prostaglandins with implications in tumour progression, and it is thus a prime target for cancer treatment (Figure 1a (ii)) [37]. In non-tumour cells, HuR is mainly localised in the nucleus, allowing miR-16 to suppress *COX-2* expression; however, the redirection of HuR to the cytoplasm in colon cancer cells leads to the stabilisation of *COX-2* mRNA through binding to an ARE element, making it less accessible to miR-16 [36]. Similarly, HuR controls receptor tyrosine kinase 2 (*ERBB-2*) mRNA in prostate cancer cells (Figure 1a (iii)) [32]. Thus, HuR interacts with the 3'-UTR of *ERBB-2* and blocks, likely through steric hindrance, the association of two miR-331-3p/RISC molecules adjacent to the HuR binding site, leading to the stabilisation of *ERBB-2* mRNAs [32]. The accumulation of *ERBB-2* in prostate cancer cells leads to the activation of signalling pathways, such as the phosphatidylinositol-3-kinase (PI3K)/AKT signalling pathway, which correlates with cancer progression and therapy resistance.

Related to HuR, the dead end 1 (DND1) protein also counteracts miRNA function by binding to U-rich regions in the 3'-UTR of mRNAs [38]. This finding from the Agami group was based on the observation that it was not only the miRNA-targeting site in mRNAs that was conserved throughout evolution but also sequences that flanked it and that these could provide a binding platform for RBPs. Such conservation has been observed between two miRNA-targeting sequences for miR-221/222 and miR-372 in the 3'-UTRs of mRNAs coding for the tumour suppressors cyclin-dependent kinase inhibitor 1B (CDKN1B, also known as p27) and large tumour suppressor kinase 2 (LATS2), respectively (Figure 1a (i)). A reporter based expression screen identified DND1 binding to this conserved region and it was shown that DND1 antagonises miRNA-mediated repression by preventing the respective miRNAs from binding to the transcripts by reducing the miRNA accessibility [38]. While the above examples concern 3′-UTRs of mRNAs, there is at least one example related to antagonistic roles within coding sequences (CDS) [39]. The inherent instability of β-transducin repeat-containing protein 1 (β*-TrCP1*) mRNA is caused by miR-183 targeting the coding sequence, however, the coding region determinant-binding protein (CRD-BP) prevents degradation of β*-TrCP1* mRNA by attenuating its miR-183-dependent interaction with Ago2 (Figure 1a (ii)) [39].

In addition to these antagonistic modes, RBPs and miRNAs can also cooperate to achieve repression of a common mRNA target, resulting in synergistic effects [25,40,41,42]. The Gorospe laboratory described strong interdependence between HuR and let-7 to repress the mRNA coding for the proto-oncogene c-Myc: as let-7 requires HuR to reduce c-Myc expression; HuR also requires let-7 to inhibit c-Myc expression (Figure 1a (iv)). This suggested a regulatory paradigm wherein HuR recruits let-7-loaded RISC to the 3'-UTR to inhibit c-Myc expression [40]. Since miRNA silencing is dependent on the accessibility of single-stranded RNA target sequences, local RNA structures could sterically hinder the binding of the miRISC complex. Such a mode of action has been experimentally confirmed for human Pumilio (PUM) protein that binds to a sequence element in the 3'-UTR of *p27* mRNA and induces a local change in the RNA structure that favours association with specific miRNAs such as miR-221/miR-222 (Figure 1a (v)) [41]. High levels of miR-221 and miR-222 are seen in many cancer cell types, which inhibit the expression of p27 and stimulate cell proliferation. In response to growth factor stimulation, PUM1 is phosphorylated, resulting in increased RNA-binding activity towards the *p27* 3'-UTR, which then favours association of miR-221 and miR-22 for efficient suppression of *p27* expression, enabling a rapid entry into the cell cycle. Likewise, a RBP-induced structural switch modulating miRNA-mediated gene expression by PUM proteins has also been described on mRNAs coding for oncogene E2F transcription factor 3 (E2F3), which is strongly repressed by the cooperative action of miR-506 and PUM1 in bladder carcinoma cells [42]. Increased expression of E2F3 is correlated with the down-regulation of mRNAs bearing a shorter 3'-UTR that does not contain the PUM1 binding site (Figure 1a (v)).

In contrast to the above studies, where specific RBPs either positively or negatively regulate miRNA targeting, a more recent study revealed the combination of both regulatory modes [43]. The pyrimidine-tract binding (PTB) protein either suppresses or enhances miRNA targeting by competitive binding on target mRNA or by altering local RNA secondary structure [43]. Particularly, it was shown that PTB directly competes with miRNAs on multiple targeting sites in the 3'-UTR of the small C-terminal domain phosphatase 1 (*SCP1*) transcript, whereas PTB binding modulates the secondary structure of the glucosamine-6-phosphate deaminase 1 (*GNPDA1*) 3'-UTR to facilitate let-7b binding (Figure 1a (v)). The interplay of the two modes, antagonistic and synergistic, with miRNA regulation could occur at different locations of the same transcript and the net additive effect may dictate the final outcome. Furthermore, PTB—better known for its role in splicing—seems to have a rather broad role in gene expression through functional interplay with the miRNA machinery. PTB, thus, has critical impact for cell fate as the reprogramming of splicing and miRNA levels induced through regulated PTB expression can drive cell fate decision towards the neuronal lineage [43].

The combinatorial control of mRNAs by ncRNA and RBPs has also been documented in differentiated neurons, where an even more complex mode of regulation has been discovered that involves two RBPs [44]. It concerns the fragile X mental retardation 1 protein (FMRP1), a well-characterised RBP with roles in mRNA transport and in the regulation of translation/ stability of mRNAs in neurons, and whose functional ablation causes Fragile X syndrome [45]. Using a transcriptome-wide approach to identify mRNA targets with iCLIP, the Ceman group identified the relationship between FMRP and Moloney Leukemia Virus 10 protein (MOV10) in modulating miRNA-mediated translational repression of several mRNAs, such as myc-associated zinc finger protein (MAZ) and MHSC1 [44]. MOV10 is an RNA helicase that unwinds GC-rich secondary structures close to miRNA binding sites in the 3'-UTR of mRNAs, enabling the assembly of miRISC complex which leads to translational repression of the mRNA target (Figure 1a (vi)). Interestingly, the resulting regulatory mode comprises two RBPs that control the action of proximal miRNAs. In the case where the FMRP binding site does not overlap with the MOV10 binding site, FMRP facilitates MOV10 binding to the mRNA and consequently, miRISC association with the mRNA for translational suppression. Conversely, in the case where the FMRP binding site overlaps with the MOV10 binding site, the respective mRNA is protected from translational suppression by miRNAs because MOV10 cannot bind to the mRNA and the formation of the miRISC complex is abolished [44].

In addition to the above examples, verified experimentally, a series of computational analyses have predicted cross-talk between RBPs and miRNA-mediated repression of human mRNAs [22,46]. For instance, global investigation of PUM mRNA targets in human cancer cells revealed that PUM recognition elements (PREs) are enriched around high-confidence miRNA binding sites in the 3'-UTRs of experimentally determined human PUM mRNA targets, suggesting interaction of PUM proteins with the miRNA regulatory systems [47]. It was also suggested that mRNAs that contain PREs in the proximity of predicted miRNA-binding sites are predicted to form stable secondary structures within their respective 3'-UTRs, and that PUM proteins are likely to be general regulators of miRNA accessibility [48]. A recent computational analysis exploring the potential cross-talk between RBPs, such as PUM proteins, and miRNA-mediated repression of human mRNAs identified a specific group of miRNA (miR-30-abcde/385-5p, miR-144, miR-376c, miR-300, miR-101, miR-221/222, and miR-410) binding sites overrepresented near PUM recognition sites in the 3'-UTR, suggesting strong cooperation to control the decay of these mRNAs [22]. Global analysis of HuR mRNA targets using photoactivatable ribonucleoside crosslinking and immunoprecipitation (PAR-CLIP) and RBP immunoprecipitation sequencing (RIP-seq) revealed the enrichment of HuR binding sites proximal to or overlapping with miRNA recognition sites [49]. Furthermore, depletion of either HuR or of 25 highly expressed miRNAs in HEK293 cells revealed that the levels of mRNA targets, with predicted overlapping miRNA and HuR binding sites, were less “upregulated” than transcripts with non-overlapping binding sites, suggesting that HuR-binding is likely to lead to the relief of miRNA-mediated regulation by competing for binding [49]. In the future, the combination of computational analysis and experimental verification will certainly reveal additional examples of interesting cross-talk between RBPs and classes of miRNAs, and it could disclose the extent of the respective modes of regulation at a global level.

As seen across these studies, the modes of cross-talk between RBPs and miRNAs are manifold and no common principle can be established. Besides the relatively “simple” models described in early studies, which referred to the competition of RBPs and miRNAs for binding sites in the mRNA resulting in antagonistic effects, the more recent studies have revealed more complex models, including synergistic effects implied by the modulation of local RNA structures to small regulatory networks that comprising additional components, e.g., additional RBPs. In this respect, while multiple examples describe the *trans*-interaction of RBPs and mRNAs to enable miRNA functions (e.g., collaboration of RBPs to enable miRISC assembly and activity), we are not aware of experimentally verified examples that describe the opposite, *trans*-interactions between miRNAs and mRNAs for controlling binding of specific RBPs to mRNAs. Interestingly, it was postulated that the assembly of miRNAs on mRNA could create 3-way RNA junctions, splinting two non-contiguous regions in the mRNA together, which could lead to the formation of a stem-loop structure targeted by certain RBPs [50]. In this model, miRNAs could interact with mRNAs and thereby change or stabilise the RNA conformation and consequentially modulate the accessibility of RBPs [50]. Furthermore, some evidence for *trans*-interactions has been obtained for long ncRNAs that could anneal with specific mRNAs in the cytoplasm forming extended regions of double-stranded RNA, which may provide an interaction platform for dsRNA-binding proteins to mediate regulatory functions [51].

## 3. Interplay of RBPs on mRNA Targets

There are numerous examples of combinatorial binding of RBPs resulting in specific splicing decisions [52,53,54]; however, much less is known about combinatorial control exerted by cytoplasmic RBPs to modulate mRNA stability or translation. The hitherto recognised events involve remodelling of RBPs activity in response to internal/ external stimuli, which are often accompanied by post-translational modifications of proteins that could affect the affinity to mRNA targets or alter their subcellular localisation [10].

Most investigations have focussed on AU-rich element binding proteins, such as HuR, which compete for binding to the same or different motifs in 3'-UTRs of particular mRNAs. An early study described the post-transcriptional regulation of eukaryotic translation initiation factor 4E (eIF4E) (the cap-binding protein), whose levels are highly elevated in many human cancers. HuR and the p42 isoform of AU-binding factor 1 (AUF1) compete for binding to the 3'-UTR of eIF4E mRNA at non-overlapping sites (Figure 1b (i)) [55]. A distinct AU-rich element in the 3'-UTR of eIF4E was found to be responsible for HuR-binding and stabilisation of the mRNA and could correlate with enhanced expression of eIF4E and HuR in malignant cancer specimens [55]. However, more common are antagonistic effects accomplished by competition at overlapping RNA binding sites [56,57]. It has been described for HuR and the ARE-binding protein Tristetraprolin (TTP), where the phosphorylation-dependent exchange of TTP and HuR provides a reversible switch between unstable and stable/efficiently translated mRNAs [56] (Figure 1b (ii)). Expression of tumour necrosis factor α (*TNF*α) mRNAs is regulated post-transcriptionally upon activation of the inflammatory response by the p38 MAPK/MK2/3 signalling pathway. Activation of this pathway leads to the phosphorylation of HuR, which becomes predominantly localised to the cytoplasm; phosphorylation of TTP decreases its affinity to ARE binding sites in the 3'-UTR of mRNAs. These phosphorylation events allow HuR to bind to the AREs routinely bound by TTP, and thus HuR further stabilises and promotes the translation of *TNF*α mRNA. Interestingly, translation of *TTP* mRNA is similarly regulated, which could shape an intrinsic feedback loop to control the extent of the inflammatory response [56]. Notably, a recent global survey of the mRNA targets for HuR and TTP unravelled thousands of overlapping TTP and HuR binding sites, suggesting that combinatorial control by these proteins likely occurs for a large number of mRNAs [58]. Likewise, a recent study showed that HuR competes with the RBP Elav-like family member 1 (CELF1) to regulate the expression of *MYC* mRNA [57]. Importantly, the antagonistic effects could play an important role in the self-renewal of the epithelium in the mammalian intestinal mucosa, which essentially depends on *MYC* activation. It could be shown that in polyamine depleted cells—the supply of polyamines controls epithelium renewalCELF1 antagonizes HuR by binding the same element in the 3'-UTR of the *MYC* transcript, causing inhibition and consequentially a decrease in MYC protein levels (Figure 1b (ii)).

There are only a few cases describing “pairs” of RBPs that cooperate and dictate a translational response or mRNA stability. One recent study described the post-transcriptional regulation of *p53* mRNA by two RBPs, the hematopoietic zinc finger protein (Hzf) and HuR, which occurs in response to signals that induce *p53* activation by the tumour suppressor protein ARF (alternative reading frame). Hzf and HuR independently associate with the 3'-UTR of *p53* mRNA, which facilitates nuclear export and translation (Figure 1b (iv)) [59]. Extensive combinatorial control by RBPs and miRNAs occurs at vascular endothelial growth factor-A (*VEGFA*) mRNAs upon external stimuli [60,61]. VEGFA has been identified as the predominant tumour angiogenesis factor in the majority of human cancers, including those of the breast, colon, lung and prostate, and thus is a major target for cancer treatment and drug development. Hypoxia, which is one of the main characteristics of the tumour microenvironment, induces *VEGFA* expression by increasing transcription, translation, and mRNA stabilisation. Post-transcriptional regulation of *VEGFA* involves different mechanisms, including the binding of cytoplasmic HuR to an ARE element in the 3'-UTR of *VEGFA* which increases mRNA stability (Figure 1b (v)) [62]; the reversal of miRNA-mediated silencing of *VEGFA* expression by RBPs [61]; and the cross-talk among different RNA-binding complexes on the mRNA [60,63]. The cross-talk occurs in a CA-rich element (CARE), localised in the 3'-UTR of *VEGFA* and it serves as a central hub for assembly and execution of regulatory events. On the one hand, the CARE is targeted by at least four different miRNAs (miR-297, miR-299, miR-567, and miR-609) and by the heterogeneous nuclear ribonucleoprotein L (hnRNP L). Since hypoxia induces the translocation of nuclear hnRNP L to the cytoplasm, this excess of hnRNP L will compete with those miRNAs for *VEGFA* mRNA binding and therefore inhibit miRNA mediated repression [61]. Additionally, hnRNP L binding induces local changes in the mRNA structure, which prevents the access of another inhibitory protein complex, the IFN-γ activated inhibitor of translation complex (GAIT) (Figure 1b (v)) [60]. Further investigation revealed that the so-called HILDA (hypoxia-inducible hnRNP L–DRBP76–hnRNP A2/B1) complex coordinates a three-element RNA switch, enabling *VEGFA* mRNA translation during concomitant hypoxia and inflammation [63].

Competition between RBPs can also occur for common mRNA features such as the m^7^G cap structure present at the 5'-end of eukaryotic mRNAs. Cap-dependent translation is conducted by the co-operation between translational factors, where eIF4E1 is the main cap binding protein [64]. Recently, Lee and co-workers described how hypoxia induces a selective competition between the two related cap binding proteins (eIF4E1 and eIF4E2) [65]. Under normal oxygen conditions (normoxia), eIF4E1 binds to the m^7^G cap at the 5'-end of mRNAs forming a complex with eIF4A, eIF3 and eIF4G and other translational factors to initiate translation. However, when cells are grown under hypoxic conditions (oxygen levels ~1%), an alternative complex is formed at the cap structure of mRNAs which contain an RNA Hypoxia Response Element (rHRE) in the 3'-UTR, such as in the epidermal growth factor receptor (*EGFR*) mRNA. This alternative complex comprises the cap-binding protein eIF4E2, oxygen-regulated hypoxia-inducible factor-2alpha (HIF-2α) and the RNA-binding protein 4 (RBM4). It was concluded that during hypoxia, cap-dependent translation of rHRE containing transcripts is eIF4E2-mediated [65]. This finding suggests that different initiation complexes may coexist in cells, competing with each other to direct the translation of subsets of mRNAs (Figure 1b (iii)).

## 4. Cross-Talk among miRNAs

It has been recognised for some time that miRNAs can work in concert to enhance the inhibition of expression of a mRNA target (Figure 1c (i)) [66,67,68]. Different pools of miRNAs may possess the ability to target a given transcript simultaneously, but in reality, this depends on the presence of the miRNAs in the same place at the same time, and miRNA expression is not uniformly distributed within different tissues and tumours [66,67,68]. For example, in prostate cancer, expression of the tensin homologue deleted on chromosome 10 (*PTEN*) transcripts is effectively repressed by the combined action of miR-106b, miR-93 and miR-25 [66]. Conversely, in breast cancer cells, *PTEN* expression is inhibited by another miRNA family namely miR-302 [69]. Since PTEN represses the activation of the PI3K/AKT/mTOR signalling pathway, decreased *PTEN* expression mediated by miRNAs leads to the activation of the PI3K/AKT/mTOR pathway, which then drives tumour progression and metastasis in many types of cancer [70]. Other related examples include the miR-182, miR-146a, and miR-146-5 that additively accomplish repression of the breast cancer-associated 1 (*BRCA1*) mRNA in breast cancer cells [67]; and the cooperation between let-7 and miR-125 miRNA families that target a set of mRNAs such as insulin-like growth factor 2 mRNA binding protein 2 (*IGF2BP2*) and pleomorphic adenoma gene-like 2 mRNA (*PLAGL2*) during the neurogenic to gliogenic transition in glial progenitor cells (Figure 1c (i)) [68].

In contrast to what has been described above, little is known about antagonistic interactions among miRNAs. Lavker and colleagues [71] have described the first example where miRNA negatively regulates another miRNA to maintain levels of a target protein. Besides demonstrating that lipid phosphatase SH2-domain-containing inositol 5-phosphatase 2 (*SHIP2*) is a target of miR-205 in epithelial cells, they observed that the corneal epithelial-specific miR-184 interferes with miR-205 to suppress *SHIP2* levels [71] (Figure 1c (ii)). Since aggressive squamous cell carcinoma (SCC) cells exhibited elevated levels of miR-205, they speculated that blockage of miR-205 activity with an antagonist or via ectopic expression of miR-184 could establish a therapeutic approach for treating aggressive SCCs.

## 5. Conclusions and Final Remarks

Although there are only a small number of experimentally established examples of combinatorial control of mRNA fates by RBPs and miRNAs, the total number of such events may be massive considering the hundreds of RBPs and thousands of miRNAs that are present in eukaryotic cells. In this regard, the recent establishment of a variety of easily accessible databases and web-based search portals provide helpful tools to perform *in silico* analyses to identify potential combinatorial events. For instance, several databases offer the possibility to scan a given RNA sequence for potential RNA binding sites of RBPs and conversely, to search for known or predicted RNA targets of a given RBP or miRNA (e.g., for RBPs: [72,73,74,75,76,77]; for miRNAs (e.g., [78,79]). This analysis can be extended to different organisms, providing information about the evolutionary conservation of particular RNA binding sites. In addition, potential binding partners related to human disease may be recognised by correlating the expression of a particular miRNA [80,81] or long noncoding (lncRNA) with human pathology (e.g., [82,83]). Using these and other bioinformatics tools, new post-transcriptional regulatory events could be deduced for further experimental testing, and possibly set a framework for the discovery of new diagnostic markers in disease.

Furthermore, it should be noted that the post-transcriptional regulators themselves undergo extensive regulation at the post-transcriptional level. RBP expression can be regulated by specific miRNAs or other RBPs, which could result in the modulation of effects exerted on the respective RBP-controlled genes (reviewed in [24,25]). It has been recognised for some time that RBPs tend to bind to messages coding for regulatory proteins such as RBPs and transcription factors (TFs) conceptualising a “regulator-of-regulator” role which is reflected by very dense post-transcriptional networks among RBPs seen in global RNA-protein interaction networks and supported by direct experimental evidence [10,84,85]. Regarding the latter, it was shown that a group of ARE-binding proteins (HuR, AUF-1, TIA1, KSRP) is controlled, at least in part, at the posttranscriptional level through a complex circuitry of self- and cross-regulatory RNP interactions [85]. Conversely, the interplay of RBPs can also directly modulate the biogenesis or localisation of miRNAs (reviewed in [24,25,86]). For instance, the Cáceres laboratory highlighted the crosstalk between heterogeneous nuclear ribonucleoprotein A1 (hnRNP A1) and KH-type splicing regulatory protein (KSRP). KSRP and hnRNP A1 compete for binding to the terminal loop of let-7a pre-miRNAs, which promotes or blocks pre-miRNA processing in somatic cells, respectively [87]. To complete the picture, it is tempting to speculate that miRNAs may also regulate the biogenesis, localisation or activity of other miRNAs. However, to our knowledge, such a strategy has not been experimentally verified.

In the light of the immense potential for combinatorial control of cytoplasmic mRNA fates, the further development of biochemical and cell-biology techniques that enable the tracking of a particular mRNA molecule in cells will be key to obtain a more comprehensive picture of when and where RBPs and ncRNAs interact with particular mRNAs for post-transcriptional control. Whereas the studies outlined above mainly approached combinatorial control from the context of the *trans*-acting factors by predicting/experimentally confirming crosstalk on mRNA targets by global or specific means, the converse approach, which involves the isolation of particular mRNAs from cells for inspection of the assembly of *trans*-acting factors, lags behind. For instance, the comparative biochemical isolation of particular mRNAs from cells or tissues at different developmental stages, upon activation of particular pathways or from pathological samples (e.g., cancer), could lead to a refined understanding of the dynamics and extent of RNP remodelling, and the elucidation of novel regulatory circuits that may be implicated in human disease.
